# Influenza Transmission in a Cohort of Households with Children: 2010-2011

**DOI:** 10.1371/journal.pone.0075339

**Published:** 2013-09-25

**Authors:** Joshua G. Petrie, Suzanne E. Ohmit, Benjamin J. Cowling, Emileigh Johnson, Rachel T. Cross, Ryan E. Malosh, Mark G. Thompson, Arnold S. Monto

**Affiliations:** 1 Department of Epidemiology, University of Michigan School of Public Health, Ann Arbor, Michigan, United States of America; 2 School of Public Health, Li Ka Shing Faculty of Medicine, The University of Hong Kong, Hong Kong Special Administrative Region, China; 3 Influenza Division, Centers for Disease Control and Prevention, Atlanta, Georgia, United States of America; Melbourne School of Population Health, Australia

## Abstract

**Background:**

Households play a major role in community spread of influenza and are potential targets for mitigation strategies.

**Methods:**

We enrolled and followed 328 households with children during the 2010-2011 influenza season; this season was characterized by circulation of influenza A (H3N2), A (H1N1)pdm09 and type B viruses. Specimens were collected from subjects with acute respiratory illnesses and tested for influenza in real-time reverse transcriptase polymerase chain reaction (RT-PCR) assays. Influenza cases were classified as community-acquired or household-acquired, and transmission parameters estimated.

**Results:**

Influenza was introduced to 78 (24%) households and transmission to exposed household members was documented in 23 households. Transmission was more likely in younger households (mean age <22 years) and those not reporting home humidification, but was not associated with household vaccination coverage. The secondary infection risk (overall 9.7%) was highest among young children (<9 years) and varied substantially by influenza type/subtype with the highest risk for influenza A (H3N2). The serial interval (overall 3.2 days) also varied by influenza type and was longest for influenza B. Duration of symptomatic illness was shorter in children compared with adults, and did not differ by influenza vaccination status.

**Discussion:**

Prospective study of households with children over a single influenza season identified differences in household transmission by influenza type/subtype, subject age, and home humidification, suggesting possible targets for interventions to reduce transmission.

## Introduction

Studies of illness in the household have a long history of providing information on age-specific incidence and characteristics of respiratory infections [1]. Data on influenza transmission from household studies carried out decades ago were vital more recently in developing models to determine national response to an influenza pandemic [2]–[4]. These models assessed the role of vaccines, antivirals and non-pharmaceutical interventions, such as school closure, in outbreak control. However, concerns have been expressed about conclusions based on these models because of known limitations in the data used to define transmission parameters [5], [6].

Households play a major role in community spread of influenza because of the frequency and intensity of contacts between household members [1]. During the recent pandemic, a number of studies of influenza transmission at the household level were carried out, many in areas outside the United States [7]–[12]. Most of these studies identified influenza index cases at contact with the health care system and then enrolled and followed eligible household members to observe transmission events. It is not certain that results from these studies can be generalized to seasonal influenza transmission. However, these studies have demonstrated the value and highlighted the need for more household studies using current laboratory methods to define illness etiology.

The extent of influenza transmission in households is estimated based on the secondary infection risk – the proportion of those exposed to index cases that are subsequently infected. Important parameters for describing influenza transmission in households include the serial interval – the time from onset of illness in an index case to onset in a secondary case, and the duration of infectiousness [2], [3], [13]. These parameters can be affected by household environment (e.g. crowding) and the age, health and vaccination status of index cases and those exposed.

We recruited and followed a cohort of 328 households with children during the 2010-2011 influenza season in Michigan, and estimated influenza transmission parameters based on real-time reverse transcriptase polymerase chain reaction (RT-PCR) confirmed outcomes. Previously these data were used to examine influenza vaccine effectiveness in preventing community and household-acquired influenza [14]. In contrast to most recent household studies [7], this study enrolled all participants before the start of influenza activity in the community, and examined influenza illnesses, whether medically-attended or not, in a season with circulation of all three influenza types/subtypes.

## Methods

### Household Eligibility, Enrollment and Data Collection

The cohort of households was derived from persons who had selected a primary health care provider from within the University of Michigan (UM) health system based in Ann Arbor Michigan; households were targeted for enrollment by direct mail [14]. Eligible households had at least four members, at least two of whom were children less than age 18 years. Adult household members provided written informed consent for participation for themselves and their children, and children age 7 to 17 years provided their oral assent. Enrollment and all follow-up activities took place at the research study site at the UM School of Public Health (UM-SPH). Health system medical records were reviewed to document the presence of health conditions considered high risk for complications of influenza [15]; electronic medical records and a statewide immunization registry were reviewed to document influenza vaccine receipt for the 2010-2011 season. Households were surveyed to collect information on household environmental factors, including crowding, humidification and exposure to tobacco smoke.

### Ethics Statement

The study was reviewed and approved by the institutional review board at the University of Michigan Medical School.

### Influenza Surveillance and Laboratory Testing

Surveillance activities were carried out from October 2010 through April 2011. Households were sent weekly email or telephone reminders to report all acute respiratory illnesses defined by two or more of the following symptoms: cough, fever or feverishness, nasal congestion, chills, headache, body aches or sore throat. Subjects with symptomatic illness attended an illness visit at the research study site within 7 days of onset and had a throat swab (or nasal swab in children <7 years) collected for influenza virus identification. Subjects were contacted by telephone 4 to 6 days after the illness visit for collection of follow-up data.

Collected specimens were tested for influenza by means of RT-PCR using the SuperScript III Platinum One-Step Quantitative RT-PCR system® and an ABI 7500 RT-PCR system platform (Life Technologies). The primers and probes used were developed by the CDC Influenza Division, and designed for universal detection of influenza A and B, and subtype identification of influenza A viruses. Laboratory tests were performed in the investigators’ respiratory virus laboratory at the UM-SPH.

### Statistical Analyses

Households were characterized by size, composition and environment, and subjects by demographics, health history and vaccination status. Influenza illnesses were characterized by type/subtype, reported symptoms, whether medically-attended or treated with antiviral medications, and by quantification of viral shedding. Illness duration was calculated as time from illness onset to reported resolution of illness symptoms; duration of symptomatic illness was used as a proxy for duration of infectiousness [8], [16].

Influenza cases were classified as household index cases (community-acquired influenza) if they were not linked by transmission from another household member. A secondary household-acquired illness was defined by transmission link to a household index case (or co-index cases) if both cases were the same influenza type/subtype and influenza onset in the secondary case occurred from 1 to 7 days after illness onset in the index case. Secondary infection risks - the proportion of those exposed to index cases that are subsequently infected - were estimated overall and for each influenza type/subtype, and examined by household environment, characteristics of index and secondary cases including age and vaccination status, and with consideration of the specimen viral loads of index cases.

Households were considered to have influenza introduced if at least one household member had community-acquired influenza (index case). Household transmission of influenza was documented if at least one household member developed influenza following exposure to a household index case, as defined above. Influenza illnesses in a household differentiated by type/subtype or separated by more than 7 days were considered separate introductions to the household from the community.

The serial interval, the time (days) from onset of illness symptoms in index cases to onset of symptoms in transmission linked secondary cases, was calculated with all transmission considered secondary to the index case [13]. Mean serial intervals were estimated overall and for each influenza type/subtype; confidence intervals around estimates were calculated using bootstrap techniques with 1000 resamples [17].

Categorical data were analyzed by Chi-square test or when necessary, Fisher exact test; continuous values were analyzed using Wilcoxon rank sum tests or ANOVA tests when comparing values across more than two categories. Survival functions were estimated and compared by log-rank test in analyses examining time to illness resolution. Statistical analyses were conducted using SAS version 9.2 (SAS Institute, Cary, NC). A *P*-value <.05 was considered to indicate statistical significance. No correction for multiple testing was considered.

## Results

### Characteristics of Households and Participants

By enrollment closure in October 2010, 328 households with 1,441 participants were enrolled. Among enrolled subjects, 58% were children less than 18 years, 11% had high risk health conditions and 60% had documentation of influenza vaccine receipt for the 2010-11 season [14]. Participant characteristics and distributions of influenza outcomes are presented in Table 1.

**Table 1:** Characteristics of all household members, those with laboratory-confirmed influenza^a^, household index cases^b^, exposed household members^c^ and secondary cases^d^: the Household Influenza Vaccine Effectiveness (HIVE) study, Ann Arbor Michigan, 2010-2011 influenza season.

Participant Characteristics	All Household Members	Laboratory-confirmed Influenza Cases^a^	Household Influenza Index Cases^b^	Exposed Household Members^c^	Household Influenza Secondary Cases^d^
	N (%^e^)	N (%^f^)	N (%^e^)	N (%^e^)	N (%^f^)
Age category					
<9 years	468 (32.5)	70 (15.0)**	50 (58.8)	84 (31.5)	14 (16.7)*
9 – 17 years	371 (25.7)	23 (6.2)	17 (20.0)	55 (20.6)	2 (3.6)
≥18 years	602 (41.8)	32 (5.3)	17 (21.2)	128 (47.9)	10 (7.8)
Sex					
Female	728 (50.5)	57 (7.8)	39 (45.9)	133 (49.8)	11 (8.3)
Male	713 (49.5)	68 (9.5)	46 (54.1)	134 (50.2)	15 (11.2)
Documented high risk health condition					
Any	162 (11.2)	19 (11.7)	14 (16.5)	26 (9.7)	4 (15.4)
None	1279 (88.8)	106 (8.3)	71 (83.5)	241 (90.3)	22 (9.1)
Documented influenza vaccine receipt					
Yes	866 (60.1)	74 (8.5)	48 (56.5)	152 (56.9)	18 (11.8)
No	575 (39.9)	51 (8.9)	37 (43.5)	115 (43.1)	8 (7.0)
Total	1,441(100)	125 (8.7)	85	267	26

* *P*-value < 0.05 from Chi-square test for independence of outcome across categories.

** *P*-value < 0.001 from Chi-square test for independence of outcome across categories.

a Includes all 125 individuals with laboratory-confirmed influenza (both index and secondary cases).

b Includes 85 index/co-index cases from the first household introductions of influenza only; 15 index/co-index cases from second household introductions of influenza were excluded.

c Includes 267 household members who were exposed to 85 index/co-index cases from the first household introductions of influenza.

d Includes 26 secondary cases resulting from the first household introductions of influenza; 4 secondary cases resulting from second household introductions of influenza were excluded.

e The percent values presented are column percentages that add to 100 for each participant characteristic.

f The percent values presented are row percentages with the cell immediately to the left as the denominator.

Household size ranged from 4 to 9 members (mean 4.4, SD = 0.7); mean household age was 22 years (SD = 5.9, range 10 to 38 years). In 55% of households more than half of subjects had documented evidence of influenza vaccine receipt. Most (78%) households reported home humidification; less than 2% reported household exposure to tobacco smoke. Household crowding was estimated based on number of persons per room with values less than the household median (0.6) indicating less crowded conditions. Household characteristics and distributions of households with influenza introduced and transmission documented are presented in Table 2.

**Table 2:** Household characteristics and the distributions of households with influenza introduced^a^ and transmission documented^b^: the Household Influenza Vaccine Effectiveness (HIVE) study, Ann Arbor Michigan, 2010-2011 influenza season.

Household Characteristics	All Households	Households with Influenza Introduced^a^	Households with Influenza Transmission^b^
	N (%^c^)	N (%^d^)	N (%^d^)
Household size: participants/household			
4 members	234 (71.3)	49 (20.9)	16 (32.7)
5 or more members	94 (28.7)	29 (30.9)	7 (24.1)
Household mean age category			
10 – 17 years	81 (24.7)	21 (25.9)*	6 (28.6)*
18 – 21 years	91 (27.7)	30 (33.0)	13 (43.3)
22 – 25 years	65 (19.8)	12 (18.5)	0 (0.0)
26 – 38 years	91 (27.7)	15 (16.5)	4 (26.7)
Households with young children (<9 yrs)			
Yes	238 (72.6)	65 (27.3)*	20 (30.8)
No	90 (27.4)	13 (14.4)	3 (23.1)
Household vaccination coverage			
None, 0%	84 (25.6)	21 (25.0)	6 (28.6)
>none, ≤50%	65 (19.8)	14 (21.5)	6 (42.9)
>50%, <100%	64 (19.5)	16 (25.0)	4 (25.0)
100%	115 (35.1)	27 (23.5)	7 (25.9)
Persons per room in home e			
≥ Median (0.6): more crowded	152 (50.0)	43 (28.3)	11 (25.6)
< Median (0.6): less crowded	152 (50.0)	32 (21.1)	11 (34.4)
Humidification of home e			
Yes	238 (78.3)	61 (25.6)	15 (24.6)
No	66 (21.7)	14 (21.2)	7 (50.0)
Exposure to tobacco smoke in home e			
Yes	5 (1.6)	1 (20.0)	0 (0.0)
No	299 (98.4)	74 (24.8)	22 (29.7)
Total	328 (100.0)	78 (23.8)	23 (29.5)

* *P*-value < 0.05 from Chi-square test for independence of outcome across categories.

a At least one household index case with community-acquired influenza.

b At least one secondary case of influenza resulting from exposure to a household index case.

c The percent values presented are column percentages that add to 100 for each household characteristic.

d The percent values presented are row percentages with the corresponding cell in the All Households column as the denominator.

e Data missing for 24 households (3 with introduction of influenza, 1 of which resulted in secondary transmission).

### Illness Surveillance and Influenza Outcomes

**Figure 1:**
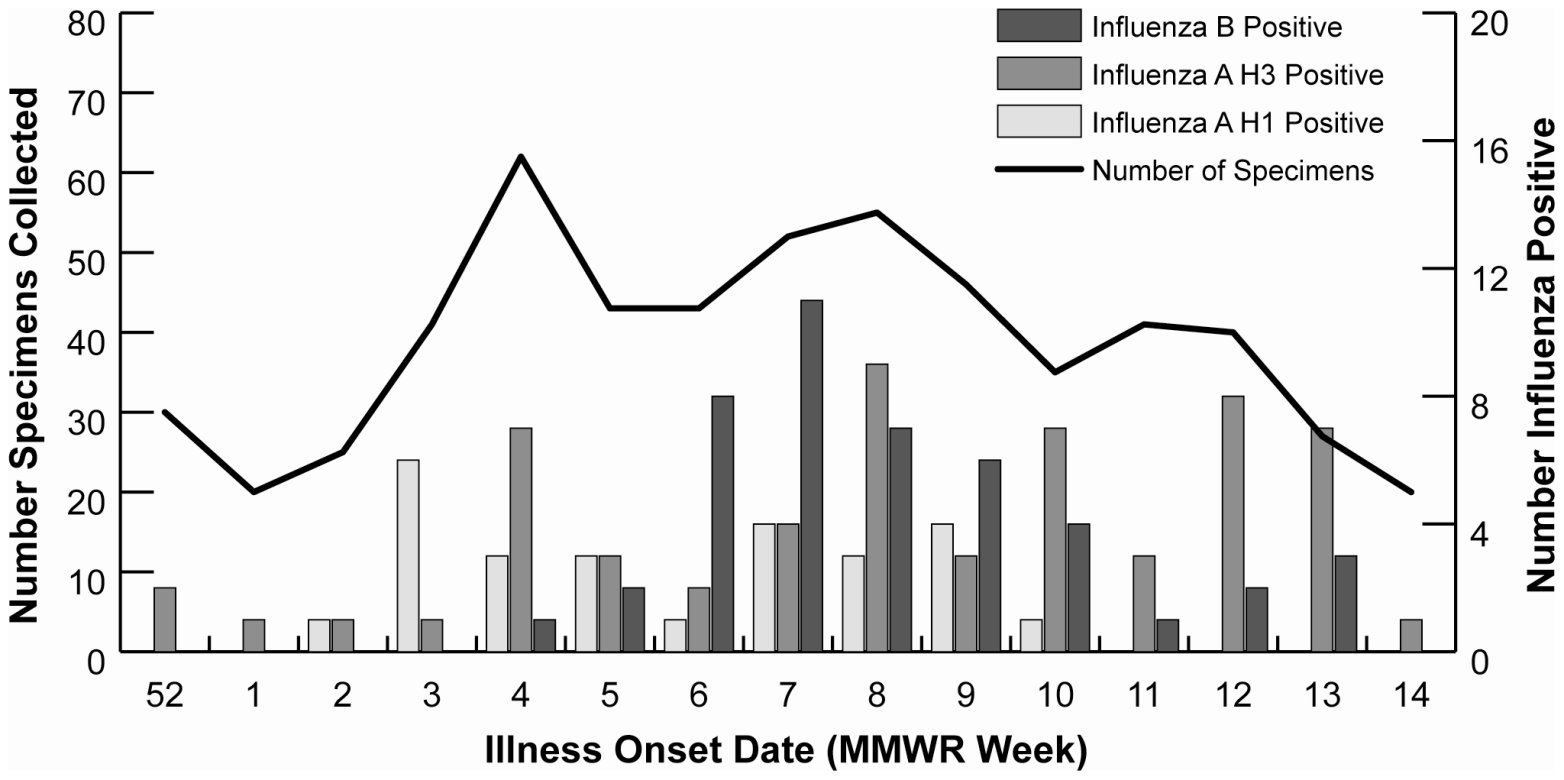
Number of specimens collected and number of influenza positive cases by week during 15 week period of influenza circulation^a,b^: the Household Influenza Vaccine Effectiveness (HIVE) study, Ann Arbor Michigan, 2010–2011 influenza season. *Footnotes:* ^a^ Week ending January 1, 2011 through week ending April 9, 2011. ^b^ 465 (32%) individuals from 193 (59%) households reported 605 acute respiratory illnesses and 580 (96%) specimens were collected. All specimens were tested for influenza viruses by reverse-transcriptase real-time polymerase chain reaction assay and 130 (22%) were determined to be positive for influenza, including 59 (45%) type A (H3N2), 44 (34%) type B, 26 (20%) type A (H1N1) pdm09 (pH1N1) and 1 (1%) type B/ type A (pH1N1) co-infection.

Influenza circulated locally between early January and early April 2011; the epidemic curve based on study surveillance is presented in Figure 1. During this period, 465 (32%) individuals from 193 (59%) households reported 605 acute respiratory illnesses and 580 (96%) specimens were collected. All specimens were tested for influenza viruses by RT-PCR and 130 (22%) were determined to be positive for influenza, including 59 (45%) type A (H3N2), 44 (34%) type B, 26 (20%) type A (H1N1)pdm09 and 1 (1%) type B/ type A (H1N1)pdm09 co-infection [14]. Antigenic testing was not performed on study specimens; however, all viral isolates assessed in another study conducted in the same community were considered antigenically similar to the vaccine strains [18].

Quantification of viral shedding was estimated based on cycle threshold (Ct) values from RT-PCR testing using previously established cut points [19]; 36 (28%) influenza positive specimens had high (Ct <25), 41 (32%) had medium (Ct 25–30) and 53 (41%) had low (Ct 31–39) viral loads. Ct values were correlated with time from illness onset to specimen collection with fewer (mean) days for those with higher viral loads (1.9 high, 2.2 medium, 2.5 low [*P* = .09]). The mean time from illness onset to specimen collection was 2.6 days for specimens testing negative for influenza, and 2.3 days for specimens testing positive for influenza (*P* = 0.12). Ct values did not significantly differ by virus type.

**Figure 2:**
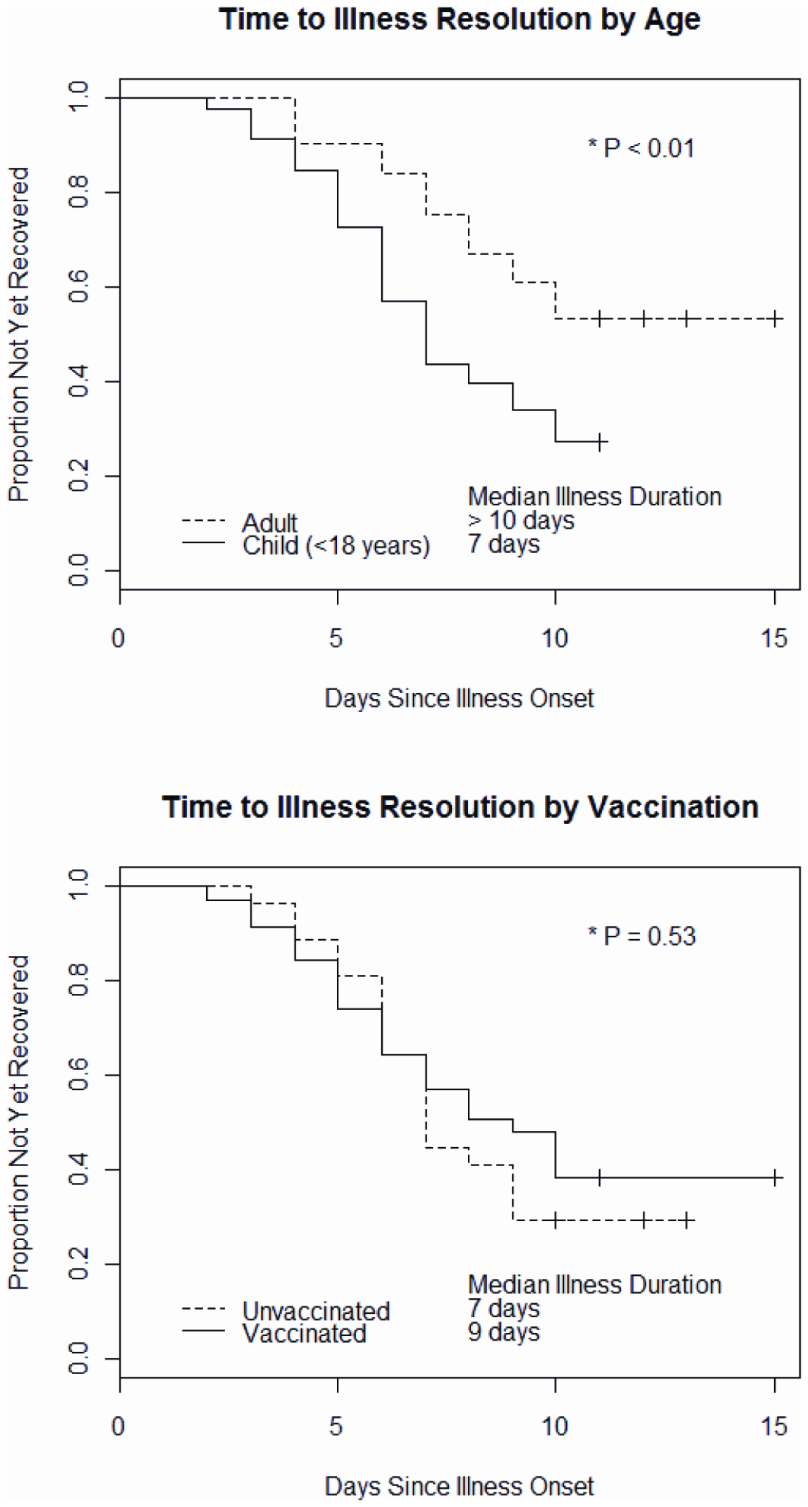
Days from influenza illness onset to resolution of symptoms by age and influenza vaccination: the Household Influenza Vaccine Effectiveness (HIVE) study, Ann Arbor Michigan, 2010-2011 influenza season. *Footnotes:* + Censored observations. * *P*-value from Log-Rank test.

Subjects with laboratory-confirmed influenza were significantly more likely to report symptoms of fever, cough, chills, body aches (all *P*<.001) and fatigue (*P* = .043) than subjects with non-influenza acute respiratory illnesses, however, median illness duration was similar (8 vs. 9 days, *P* = 0.93). Forty-two (32%) influenza cases were medically-attended based on medical record review and three cases (2%) were treated with antiviral medications; two of the three treated cases were children, both had high risk health conditions. Median illness duration significantly varied by age category with shorter duration of symptoms among children compared with adults (7 days vs. >10 days, *P* = .01) (Figure 2); illnesses were not yet resolved for 52% of influenza cases at time of final illness follow-up. Illness duration did not significantly vary by influenza type/subtype, or based on presence of high risk health condition or the influenza vaccination status (9 days vaccinated cases vs. 7 days unvaccinated cases, *P* = .53) (Figure 2) of case subjects.

### Influenza Risk by Household and Participant Characteristics

Influenza was identified in 78 (24%) households and 125 (9%) individuals, including 5 individuals with two separate influenza infections (all >14 days apart; 3 of 5 had both influenza type A and type B infections). Younger households (those with mean age <22 years) and households with young children (age <9 years) were more likely to have influenza introduced (Table 2). None of household size or crowding, household vaccination coverage, humidification or exposure to tobacco smoke was significantly associated with likelihood of influenza introduction.

Overall influenza infection risks significantly varied by subject age category (*P*<.001) and were highest among children age <9 years (15.0%) and lowest among adults (5.3%) (Table 1). There were no significant differences in influenza infection risk by sex, presence of high risk health condition or documented influenza vaccine receipt.

### Household Transmission of Influenza

There were 91 total introductions of influenza to 78 households by 100 index or co-index community-acquired cases. Thirteen households had two introductions each (differentiated by influenza type/subtype or time); co-index cases (identical illness onset dates) were identified in eight introduction events – seven with two index cases and one with three index cases.

Household transmission of influenza was demonstrated in 23 (29%) households as a result of first (n = 20) or second (n = 3) influenza introductions, with 30 cases of secondary household acquired influenza identified. Seventeen introductions each produced one secondary case, five each produced two secondary cases, and one produced three secondary cases. The likelihood of household transmission was not associated with household size, vaccination coverage, crowding or exposure to tobacco smoke, but was more likely in households with younger mean household age (<22 years)(*P*<.05) and households reporting no home humidification (*P* = 0.10) (Table 2).

Multiple introductions of influenza to some households complicated examination of the characteristics of index cases and those exposed, and estimation of secondary infection risks, as some subjects were both index cases and exposed household members. As a result only the first household introductions of influenza were considered here. First introductions to 78 households, committed by 85 index or co-index cases, exposed 267 household members and resulted in 26 secondary household-acquired cases in 20 households for a secondary infection risk of 9.7% [26/267].

Characteristics of household index cases, exposed household members and household secondary cases are presented in Table 1. Household index cases were most likely to be young (<9 years) children (59%) and equally likely to be older children (20%) or adults (21%); 17% of index cases had a high risk health condition and 57% had documented influenza vaccine receipt. The secondary infection risk among exposed household members significantly varied (*P* = .02) by age category of exposed subjects; risk was highest among young children (16.7%), lowest among older children (3.6%) and intermediate for adults (7.8%). Secondary infection risks were higher among exposed household members with high risk conditions and those with documented influenza vaccine receipt, but not significantly.

Secondary infection risks by characteristics of household index cases are presented in Table 3. Secondary risks did not significantly vary based on the age, health or vaccination status of household index cases. Household members exposed to index cases with high or medium specimen viral loads had a significantly higher secondary infection risk than those exposed to index cases with low specimen viral loads. Secondary infection risks varied significantly by influenza type/subtype with the highest secondary risk for influenza A (H3N2) [15.3%] and the lowest for influenza A (H1N1)pdm09 [2.9%]; influenza B was intermediate [7.7%].

**Table 3:** Secondary infection risks by characteristics of household influenza index cases^a^: the Household Influenza Vaccine Effectiveness (HIVE) study, Ann Arbor Michigan, 2010-2011 influenza season.

Characteristic of Index Case	Number of Household Contacts Exposed	Number of Secondary Cases	Secondary Infection Risk
Age <9 years			
Yes	154	17	11.0%
No	113	9	8.0%
Male			
Yes	146	16	11.0%
No	121	10	8.3%
Documented High Risk Health Condition			
Yes	51	7	13.7%
No	216	19	8.8%
Documented influenza vaccine receipt			
Yes	164	19	11.6%
No	103	7	6.8%
RT-PCR Ct ≤ 30^b^			
Yes	144	19	13.2%*
No	123	7	5.7%
Influenza type (subtype)			
A (H3N2)	111	17	15.3%**
A (pH1N1)^c^	68	2	2.9%
B^c^	91	7	7.7%
Total	267	26	9.7%

* *P*-value < 0.05 from Chi-square test for independence of outcome across levels of categorical exposure.

** *P*-value < 0.001 from Chi-square test for independence of outcome across levels of categorical exposure.

a Only the first household introductions of influenza are considered here; 15 index/co-index cases, and 4 secondary cases were excluded.

b Reverse-transcriptase real-time polymerase chain reaction (RT-PCR) assay cycle-threshold (Ct) values are inversely related to viral load (i.e. lower Ct values are associated with higher viral load).

c One index case had an influenza A (pH1N1) and influenza B coinfection. Household contacts exposed to this index case are included in both the influenza A (pH1N1) and influenza B secondary infection risk estimates.

The serial interval was calculated overall and by influenza type/subtype, with secondary cases resulting from both first and second influenza introductions to households considered. The mean serial interval was 3.2 days overall (Table 4). Serial intervals were similar for influenza A (H3N2) and A (H1N1)pdm09 (2.5 vs. 2.8 days), but significantly longer for influenza B (4.9 days, *P* = .02).

**Table 4:** The serial interval for cases of household influenza transmission overall and by influenza type and subtype: the Household Influenza Vaccine Effectiveness (HIVE) study, Ann Arbor Michigan, 2010-2011 influenza season.

	Number of Secondary Influenza Cases^a^	Mean Serial Interval^b^	95% CI^c^
Any Influenza	30	3.2	2.4 – 3.9
A (H3N2)	17	2.5	1.8 – 3.3
A (pH1N1)	5	2.8	1.3 – 5.0
B	8	4.9	3.3 – 6.3

a All 30 secondary influenza cases from resulting from all household introductions of influenza were included here.

b Mean serial interval: mean days between onset of illness symptoms in index cases to onset of symptoms in transmission linked secondary cases.

c 95% confidence intervals (95% CI) were calculated using bootstrap techniques with 1000 resamples.

## Discussion

Our household study was originally designed and statistically powered to estimate influenza vaccine effectiveness in preventing community acquired influenza [14], with a secondary objective of examining influenza transmission parameters. In contrast to other studies carried out to describe influenza transmission, we utilized a cohort design with long term follow-up of households, and required that households had at least four members including at least two children. This strategy increased the likelihood of studying households with influenza introduction and the opportunities for examining factors, including both subject and household characteristics, associated with transmission. Influenza outcomes were laboratory-confirmed and illnesses of any severity considered.

It has been estimated that approximately one-third of all influenza transmission occurs within households [1]. The likelihood of infection following exposure in the household is thought to be far greater than in the community, because of the frequency and intensity of contacts [20]. For this reason, interventions targeting household transmission may be particularly effective in reducing the impact of influenza outbreaks. Because much of the existing knowledge about household transmission has come from studies carried out decades ago or during the recent pandemic, current estimates of household transmission parameters are of particular value for planning and modeling seasonal influenza interventions.

Secondary infection risks describe the extent of influenza transmission in the household setting and estimated values may vary by study design, influenza type/subtype, the infectiousness of index cases and the susceptibility of contacts [7]. Our estimated secondary infection risk (9.7%) falls in the mid-range of estimates reported from studies with similar designs (4%–17%) [10]–[12], [21]–[22]; however, we may have underestimated the actual value. Studies, such as ours, that test only symptomatic household members generally report lower estimates than those testing all household contacts of symptomatic cases [7]; however, our case definition was designed to facilitate collection of specimens from even mild illnesses. Further, co-index cases were identified in 8 of 91 household introductions of influenza. This could have resulted by chance, common exposure, or inaccurate reporting of illness onset dates. If reported onset dates were inaccurate, some secondary cases may have been misclassified as co-index cases resulting in a lower estimate. Finally, asymptomatic or sub-clinical infections were not considered. Estimates of the proportion of infections that are asymptomatic vary and their contribution to transmission is unclear [9], [16], [23].

Consistent with previous reports [8], [11]–[12], [21], we found household index cases were most likely to be young children (<9 years), and secondary infection risks were highest among young (<9 years) contacts. This suggests that interventions designed to interrupt household transmission may be particularly effective if they intervene on contacts with or between children (e.g. isolation). We also observed that secondary infection risks were higher among adult contacts (7.8%) of index cases than among exposed older children (3.6%) (*P* = 0.35); in subsequent study years, we are examining care-giving behaviors as a possible explanation for this finding. Interestingly, households with influenza introduced were less likely to experience secondary transmission if they reported home humidification, but this effect was not statistically significant. A relationship between humidity and influenza transmission has been previously reported [24], and this finding suggests another possible intervention to reduce household transmission.

Secondary infection risks significantly varied by influenza type/subtype with the highest risk among those exposed to type A (H3N2) [15.3%], even though risks of community-acquired infection (A (H3N2) [2.9%], A (H1N1)pdm09 [1.5%], and B [2.6%]) were similar for all types/subtypes [14]. Variation in secondary risk by influenza type/subtype could be due to differences in levels of immunity among household contacts [7]–[8]. Prior to the 2010–2011 season, subjects were likely exposed to both waves of the 2009 H1N1 pandemic, influenza A (H3N2) had not been the dominant circulating strain since the 2007–2008 season, and influenza B had circulated at relatively low levels. Unfortunately, we were unable to judge immune susceptibility to influenza infection in this season. In subsequent study years, we are collecting blood specimens from participants at multiple time points; inclusion of serologic assessments of immunity should allow calculation of infection risks among subjects with different levels of susceptibility. Serologic data may also permit estimation of the number of asymptomatic (or unreported) infections, although serologic confirmation of infection in the vaccinated is problematic [25], and the sensitivity of the traditional four-fold rise to determine infection remains uncertain [26].

Previous estimates of the serial interval for influenza have ranged from 1 to 4 days, with most estimates falling between 2.5 to 3.5 days [7], [13], [27]. Our observed household serial interval (overall 3.2 days) was consistent with these prior estimates. We considered all secondary cases to be transmitted from the index case, with no tertiary transmission. Failure to consider chains of transmission can result in overestimation of the serial interval; however, here the mean serial interval was similar if potential tertiary transmission was considered (3.1 vs. 3.2 days). Based on a relatively small number of secondary cases, we found the serial interval for influenza B (4.9 days) was significantly longer than for influenza A (H3: 2.5 days, H1: 2.8 days). Serial intervals can be influenced by contact patterns (number, intensity, and duration), infectivity of index cases, and susceptibility of contacts. The longer serial interval for influenza B is consistent with lower infectivity for influenza B, and the relatively higher vaccine effectiveness against influenza B estimated for the 2010–2011 season [14], [18].

Illnesses were followed with a single follow-up contact 4 to 6 days after the illness visit (which occurred up to 7 days after illness onset) and at that time, half of the influenza cases noted their illnesses were not yet resolved. Our estimate of median illness duration of 8 days is similar to previously reported estimates ranging from 7 to 11 days [20], [25], [28]. The relationship between reported duration of illness symptoms and the duration of infectiousness is unclear. Viral shedding as measured by RT-PCR has been shown to correlate with illness symptoms [8], [16]; however, the infectious period has also been estimated to be days shorter than the duration of symptoms [20]. Our findings that adults had relatively longer reported illness duration will be investigated further in subsequent study years with more thorough follow-up to capture resolution of all illnesses. Our finding that illnesses in which influenza was identified were more likely than non-influenza respiratory illnesses to be characterized by fever, cough, chills, body aches and fatigue is similar to previous reports [29]–[31].

In our descriptive analysis, household and subject characteristics associated with transmission parameters were not examined in multivariable adjusted models. The value of using traditional multivariable statistical models was limited by small sample size and complicated by factors such as influenza introduction by co-index cases, multiple introductions of influenza to a household from the community and varying risk status as household infections occurred. More complex models, including dynamic systems models [20], are necessary to fully utilize these data to make inferences about influenza transmission and transmission parameters.

Our objectives here were to describe transmission in households with children, and examine factors and parameters that affected transmission. Enrolled households were highly vaccinated and as previously reported, we found no evidence of vaccine effectiveness in preventing household-acquired influenza [14]. Further, our findings of high household transmission risk for influenza A (H3N2), despite similar community risks across influenza types/subtypes, are consistent with the poor vaccine effectiveness demonstrated against A (H3N2) that season [14], [18], [32]. These findings highlight the need for improved vaccines, but also emphasize the potential value of non-pharmaceutical interventions in reducing household transmission of influenza.
